# Identifying Sentiment of Hookah-Related Posts on Twitter

**DOI:** 10.2196/publichealth.8133

**Published:** 2017-10-18

**Authors:** Jon-Patrick Allem, Jagannathan Ramanujam, Kristina Lerman, Kar-Hai Chu, Tess Boley Cruz, Jennifer B Unger

**Affiliations:** ^1^ Keck School of Medicine of USC Los Angeles, CA United States; ^2^ University of Southern California Los Angeles, CA United States; ^3^ University of Pittsburgh Pittsburgh, PA United States

**Keywords:** hookah, waterpipe, Twitter, social media, bots, big data, sentiment

## Abstract

**Background:**

The increasing popularity of hookah (or waterpipe) use in the United States and elsewhere has consequences for public health because it has similar health risks to that of combustible cigarettes. While hookah use rapidly increases in popularity, social media data (Twitter, Instagram) can be used to capture and describe the social and environmental contexts in which individuals use, perceive, discuss, and are marketed this tobacco product. These data may allow people to organically report on their sentiment toward tobacco products like hookah unprimed by a researcher, without instrument bias, and at low costs.

**Objective:**

This study describes the sentiment of hookah-related posts on Twitter and describes the importance of debiasing Twitter data when attempting to understand attitudes.

**Methods:**

Hookah-related posts on Twitter (N=986,320) were collected from March 24, 2015, to December 2, 2016. Machine learning models were used to describe sentiment on 20 different emotions and to debias the data so that Twitter posts reflected sentiment of legitimate human users and not of social bots or marketing-oriented accounts that would possibly provide overly positive or overly negative sentiment of hookah.

**Results:**

From the analytical sample, 352,116 tweets (59.50%) were classified as positive while 177,537 (30.00%) were classified as negative, and 62,139 (10.50%) neutral. Among all positive tweets, 218,312 (62.00%) were classified as highly positive emotions (eg, active, alert, excited, elated, happy, and pleasant), while 133,804 (38.00%) positive tweets were classified as passive positive emotions (eg, contented, serene, calm, relaxed, and subdued). Among all negative tweets, 95,870 (54.00%) were classified as subdued negative emotions (eg, sad, unhappy, depressed, and bored) while the remaining 81,667 (46.00%) negative tweets were classified as highly negative emotions (eg, tense, nervous, stressed, upset, and unpleasant). Sentiment changed drastically when comparing a corpus of tweets with social bots to one without. For example, the probability of any one tweet reflecting joy was 61.30% from the debiased (or bot free) corpus of tweets. In contrast, the probability of any one tweet reflecting joy was 16.40% from the biased corpus.

**Conclusions:**

Social media data provide researchers the ability to understand public sentiment and attitudes by listening to what people are saying in their own words. Tobacco control programmers in charge of risk communication may consider targeting individuals posting positive messages about hookah on Twitter or designing messages that amplify the negative sentiments. Posts on Twitter communicating positive sentiment toward hookah could add to the normalization of hookah use and is an area of future research. Findings from this study demonstrated the importance of debiasing data when attempting to understand attitudes from Twitter data.

## Introduction

The popularity of hookah (or waterpipe) use is increasing among youth and young adults in the United States and elsewhere [1]. This increase will have consequences for public health because hookah use has similar health risks to that of combustible cigarette use [2,3]. Hookah is often perceived as safer than cigarettes [4] and is subject to less regulation [5]. For example, hookah is offered in a variety of flavors and often receives exemptions on smoking bans in bars and nightclubs. As hookah use increases in popularity, social media data (Twitter, Instagram) can be used to capture and describe the social and environmental context in which individuals use, perceive, discuss, and are marketed this tobacco product [6]. These data may allow people to organically report on their sentiment toward tobacco products like hookah unprimed by a researcher, without instrument bias, and at low cost [7].

In a recent study analyzing hookah-related posts on Instagram, researchers reported that hookah was often cross-promoted with alcohol by nightclubs, bars, restaurants, and hookah lounges [8]. Instagram posts often showed hookah use in social settings as well as stylized and elaborate waterpipes [8]. Twitter posts have previously been used to study hookah. Krauss et al coded a sample of 5000 hookah-related tweets and reported that 87% of the tweets in their sample normalized or made hookah smoking appear common or portrayed positive experiences with smoking hookah [9]. The authors also noted that only 7% of tweets were against hookah or discouraged its use [9]. Grant and O’Mahoney coded a sample of 4439 tweets and reported that 59% of tweets were positive about hookah use, with 3% negative, 21% lacking sentiment, and 17% unclassifiable [10]. Myslín et al analyzed a sample of 7362 tobacco-related tweets, some referencing hookah, and found that sentiment toward tobacco was overall more positive (46% of tweets) than negative (32%) [11].

In this study, we demonstrate the feasibility of a Twitter-based “infoveillance” [6] methodology to document sentiment of hookah-related posts. This study also relied on machine learning to debias the data so that Twitter posts were reflective of sentiment of legitimate human users and not of social bots or marketing-oriented accounts that would possibly provide overly positive or overly negative sentiment [12-14]. As Allem and Ferrara described, “Studies using tweets and that aimed at gaining insights to individual-level attitudes and behaviors are now faced with data with substantial bias and noise. Any results drawn upon this data and not preprocessed with de-noising techniques lose validity and significance” [14]. To demonstrate the importance of debiasing Twitter data, comparisons were made between corpuses of tweets that included and excluded social bots. Findings from this study can inform tobacco control, demonstrate the utility in using social media data in understanding attitudes, and demonstrate the importance of debiasing Twitter data when attempting to understand attitudes.

## Methods

Data were obtained from Twitter’s Streaming Application Program Interface (API) based on Twitter4J libraries, an open source database of java language used to analyze data from the API. Software was written to automate this process. Tweets posted between March 24, 2015, and December 2, 2016, were collected. The root terms used to collect the sample of tweets were hookah(s) or hooka(s) or sheesha(s) or shisha(s) or sesh(s). The root terms could have appeared in the post or in an accompanying hashtag, for example, hookah or #hookah. While the word waterpipe is commonly used in academic papers and presentations to refer to hookah, it is uncommon for individuals to use this term on social media and it was thus not included in this study [15].

The root terms used to collect tweets during the study period resulted in an initial corpus of tweets (N=986,320). However, Twitter has quickly become subject to third party manipulation where social bots, or computer algorithms designed to automatically produce content and engage with legitimate human accounts on Twitter, are created to influence discussions and promote specific ideas or products [12-14]. Social bots are meant to appear to be everyday individuals operating Twitter accounts that are complete with metadata (name, location, pithy quote) and a photo/image. Social bots make indiscriminate references to an array of content while at the same time perpetuating select conversations giving the appearance that a specific topic is more prominent than it actually is offline. In order to debias the data, select features—(1) the timing of tweets (periodic and regular), (2) spam or not (if the post contains known spam), and (3) ratio of tweets from mobile versus desktop (as compared to the average human Twitter user)—were used to differentiate between legitimate human accounts and social bots following the methods described by Chu et al [16].

**Figure 1:**
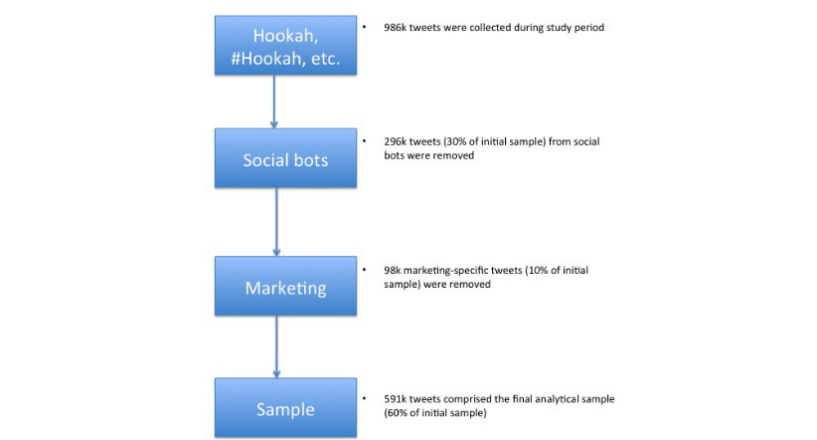
Flowchart of how the analytic sample was derived.

Additionally, certain criteria such as information diffusion patterns (based on Twitter’s message forwarding function known as “retweets” or mentions), friend features (ratio of followers to followees), content (frequency of nouns/verbs/adverbs in a tweet in comparison to a legitimate human account), and sentiment features (derived from emotion scores) following Ferrara et al’s methods were combined to arrive at a single score that indicated if a Twitter account was a social bot or not [13]. Exactly 296,338 (30.04%) of the initial posts were determined to be from social bots and were removed from the corpus. Since marketing-specific tweets would not reflect public sentiment, they were manually removed based on occurrence of certain keywords. For example, “1100mah” (strength of hookah) was one such term commonly found in marketing posts. The number of marketing-specific tweets removed was 98,190, resulting in the final analytical sample of 591,792 tweets (Figure 1).

After debiasing the data, machine learning methods for natural language processing (NLP) were used to identify sentiment of tweets. NLP primarily involves either rule-based reasoning or automated inference logic, and in this study we used both approaches. Rule-based reasoning involves explicit rules to identify a sentence as “positive” or “negative.” Specific words or phrases were labeled and put on a spectrum ranging from -4 to 4, following the method of Hutto and Gilbert [17]. For example, a negative word such as “horrible” has a value of -2.5 and a positive word such as “wonderful” has a value of 2.7. Words and phrases were added to a list, and based on their occurrence in a tweet, an overall sentiment score was calculated for each tweet.

In addition to these explicit values, certain grammatical structures in English were exploited to add to or subtract from the overall sentiment scores. Capital letters, punctuation like an exclamation point, or degree modifiers such as “extremely” or “very” followed by a positive or negative word were considered twice. For example, “very happy smoking hookah” considered the word “happy” occurring twice because of the degree modifier “very” preceding it. Additionally, grammatical features such as the use of the word “but” show a shift in polarity of the words following it. By considering the shift in polarity in a statement, we could capture n-gram features (an n-gram is a phrase with n words). For example, we could identify the sentiment from the sentence “the hookah here isn’t really all that great” as negative.

To determine the performance of the rule-based reasoning model, the sentiment output of the tweets was analyzed. The rule-based reasoning sentiment analysis provided an *F* score of 0.96. The *F* score is a measure of a test’s accuracy that reflects the balance achieved between identifying cases correctly and recalling a high number of correct cases. In NLP, it considers both “Precision” or “P”, which answers the question, “What portion of what you found was ground truth (eg, what percent of true cases were categorized accurately)?” and “Recall” or “R”, which answers the question, “What portion of the ground truth did you recover (eg, what percentage of true cases did you recall?” The *F* score is the harmonic mean of P and R=[(2*P*R)/(P+R)].

The rule-based reasoning model was considered as a reference point, or baseline model, to inform a model based on a support vector machine (SVM) algorithm. Combining a manual rule‒based method with an automated one like SVM provided us with a more generalized solution for sentiment analysis. In the implementation of this algorithm, the model was trained on the SemEval [18] and the ISEAR [19] emotion datasets, and on an emotion-tagged tweet corpus that has been established in past studies to identify sentiments [20] with cross-validation. This comparison served to validate our results. Based on the frequency of occurrence of n-gram phrases from the SemEval [18] and ISEAR [19] datasets, the tweet corpus (test data) was tagged with the appropriate sentiments and scored. The aggregate emotion score of all tweets was calculated as a linear combination of the given emotion scores for individual tweets.

In order to utilize an SVM model, the data have to be prepared as follows. (1) First the tweets are cleaned to permit uniform analysis using a process called tokenization that involves removing uniform resource locators (URLs) and unrecognizable UTF-8 encoding forms from the text of tweets along with stop words such as “the” and “an,” which frequently occur in spoken and written language but do not convey sentiment. Hypertext markup language (HTML) tags were replaced by whitespace, and then a pattern-matching algorithm was used to recognize any trailing URL patterns that remained in the text. (2) This preprocessing step also included allowing the model to recognize emoticons (eg, ☺), slang, and aggravated language usage (eg, “this is grrrrrreat!”). Emoticons were unified so that all types of smiley faces were considered one, while all types of sad faces were considered one. Slang was corrected and punctuation was fixed, for example, “don’t” and “dont” were treated as one. Gerunds or stemming in NLP were handled by the program Porter’s Stemmer [21], and elongations were removed to correct for aggravated language usage. (3) Part of Speech (POS) tagging was done using CoreNLP’s POS tagger [22], in order to identify verbs, nouns, adjectives, and prepositions. Russell’s range of 20 different emotions was used to tag tweets from unpleasant to pleasant on a negative to positive scale [23]. The SVM algorithm was used to generate feature vectors and arrive at a sentiment score for each tweet (*F*=0.90).

In order to quantify and compare the impact of bias from social bots, we reduced the 20 levels of emotions down to 5 primary emotions (anger, fear, joy, sadness, disgust) as described by Bradley et al [24]. We then report the specific probabilities that a tweet will have a specific sentiment from each corpus of tweets directly comparing the corpus of tweets from legitimate human accounts to that of tweets from the corpus with human accounts and social bots.

## Results

**Figure 2:**
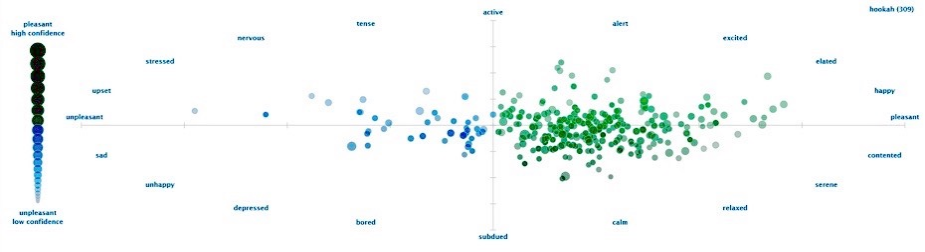
Tagged tweets showing range of emotions from unpleasant to pleasant.

**Figure 3:**
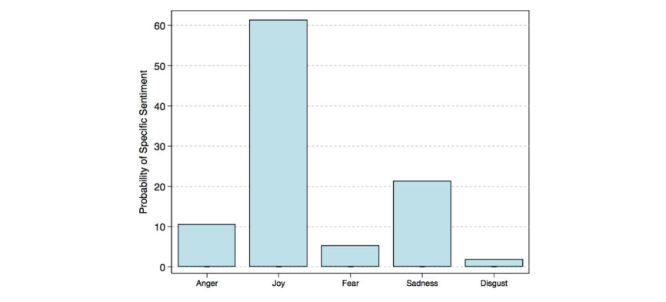
Probability of one tweet’s specific sentiment from the debiased (or social bot free) corpus of tweets.

**Figure 4:**
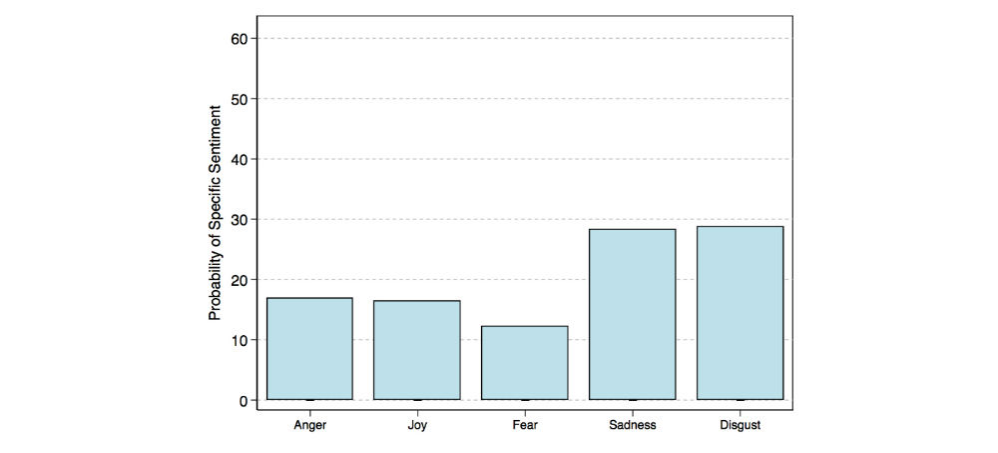
Probability of one tweet’s specific sentiment from the biased corpus.

A majority of tweets 352,116 (59.50%) were classified as positive while 177,537 (30.00%) were classified as negative (Figure 2), and 62,139 (10.50%) neutral. Among all positive tweets, 218,312 (62.00%) were classified as highly positive emotions (top right quadrant of Figure 2), for example, active, alert, excited, elated, happy, and pleasant. The remaining 133,804 (38.00%) positive tweets were classified as passive positive emotions (bottom right quadrant of Figure 2), for example, contented, serene, calm, relaxed, and subdued. Among all negative tweets, 95,870 (54.00%) negative tweets were classified as subdued negative emotions (bottom left quadrant of Figure 2), for example, sad, unhappy, depressed, and bored. The remaining 81,667 (46.00%) were classified as highly negative emotions (top left quadrant of Figure 2), for example, tense, nervous, stressed, upset, and unpleasant. The results change drastically once tweets from social bots are in the corpus. Among the 888,130 tweets (tweets from individual accounts and social bots but excluding marketing), 324,331 (36.52%) were classified as negative, while 300,660 (33.85%) were classified as positive and 263,139 (29.63%) neutral. When reducing the 20 levels of emotion down to 5 primary emotions, the probability of any one tweet reflecting joy was 61.30% from the debiased (or bot free) corpus of tweets (Figure 3). In contrast, the probability of any one tweet reflecting joy was 16.40% from the biased corpus (Figure 4).

## Discussion

### Principal Findings

Social media data provide researchers the ability to understand public sentiment and attitudes by listening to what people are saying in their own words. By using Twitter data, we had the ability to document and describe individuals’ sentiment toward a tobacco product without being primed by a researcher, allowing for spontaneous comments to emerge. We found that the majority of hookah-related posts on Twitter conveyed positive sentiment. Positive posts on Twitter may serve as a screener indicating that an individual is in need of tobacco-related education to reduce misconceptions about a product or to reduce positive social norms about that product. Tobacco control programmers in charge of risk communication may consider targeting individuals posting positive messages about hookah on Twitter or designing messages that amplify the negative sentiments. Twitter may be used to bolster the reach and delivery of health information that communicates the risk of tobacco use [25-27], and social media interventions hold promise for getting people to consider stopping smoking [28].

In comparison to earlier studies that relied on sample sizes small enough to reasonably code by hand [9,10] during brief study periods [9-11], we collected hundreds of thousands of Twitter posts continuously over 20 months that pertained to hookah. We also trained machine learning algorithms to automatically determine sentiment of these posts following Russell’s [23] range of emotions to predict a sentiment score on 20 different emotions on a two-dimensional scale demonstrating greater depth in sentiment classifications. This range of emotion reflects a greater systematic assessment of sentiment than prior work that relied on dichotomous classification based on subjective individual judgment [9-11]. While Twitter posts referenced hookah use in a happy, excited, and alert fashion, posts also referenced hookah use in more passive ways. These nuances may reflect the social and environmental contexts in which hookah use often takes place. Posts on Twitter conveying positive sentiment toward hookah could add to the normalization of hookah use and is an area of future research.

While in this study, 30% of hookah-related posts contained negative sentiment, earlier studies found that negative posts were relatively rare. For example, Grant et al reported 3% of Twitter posts in their data conveyed negative sentiment [10]. This discrepancy could be a result of the sample size (n=4439) or short study period (1 week) in prior work [10]. Alternatively, findings from previous work may have been biased due to social bots in the Twitter data [14].

In this study, 296,338 Twitter posts were removed because they were found to be from social bots and would not accurately reflect individual sentiment toward hookah use. This number reflects 30% of the initial data that comprised the corpus of tweets. Removing social bots from the analytical corpus had marked effects on the results. The bot free corpus reflected that the overall sentiment was positive (eg, 59.5% of tweets were positive). The corpus that included bots and human accounts together reflected that only 33.9% of tweets were positive. While sentiment in tweets is only one of many possible ways bias from social bots can distort Twitter data, the findings from this study highlight the importance of debiasing data collected from Twitter in order to uncover the rich and nuanced information available to health researchers relying on social media data and aimed at understanding public attitudes [14].

### Limitations

Data relied on Twitter’s Streaming API, which prevented us from collecting tweets from private Twitter accounts. As a result, findings may not represent the sentiment toward hookah from individuals with private accounts. The result that more tweets were found to convey a positive sentiment than a negative one should be considered circumstantial and not absolute. While we identified the overall sentiment of posts, we did not track individual users to see whether their posts about hookah change over time. Additionally, a post classified as negative does not necessarily mean the person dislikes hookah. Rather, it means the post had more words conveying negative sentiment than positive. The method used in this study for bot detection is not a perfect system but scores a detection accuracy above 95% suggesting biases from inappropriate removal of legitimate accounts is minimal [13].

### Conclusion

Despite these limitations, this study demonstrated the utility in using social media data to understand public attitudes that may influence acceptance of tobacco products such as hookah and defined types of positive and negative attitudes that could be incorporated into public health media campaigns to reduce acceptance of hookah. It also illustrated the importance of debiasing Twitter data so that posts reflect sentiment of legitimate human users and not of social bots or marketing-oriented accounts that could possibly provide overly positive or overly negative sentiment of hookah. Findings should spur efforts to better understand the consequences of hookah-related discussions on Twitter as an informative tool in planning tobacco control efforts.

## Abbreviations

API: application program interface
HTML: hypertext markup language
NLP: natural language processing
POS: part of speech
SVM: support vector machine
URL: uniform resource locator

## References

[1] Salloum RG, Asfar T, Maziak W (2016). Toward a Regulatory Framework for the Waterpipe. Am J Public Health.

[2] Maziak W (2011). The global epidemic of waterpipe smoking. Addict Behav.

[3] El-Zaatari ZM, Chami HA, Zaatari GS (2015). Health effects associated with waterpipe smoking. Tob Control.

[4] Heinz AJ, Giedgowd GE, Crane NA, Veilleux JC, Conrad M, Braun AR, Olejarska NA, Kassel JD (2013). A comprehensive examination of hookah smoking in college students: use patterns and contexts, social norms and attitudes, harm perception, psychological correlates and co-occurring substance use. Addict Behav.

[5] Primack BA, Hopkins M, Hallett C, Carroll MV, Zeller M, Dachille K, Kim KH, Fine MJ, Donohue JM (2012). US health policy related to hookah tobacco smoking. Am J Public Health.

[6] Eysenbach G (2011). Infodemiology and infoveillance tracking online health information and cyberbehavior for public health. Am J Prev Med.

[7] Ayers JW, Althouse BM, Dredze M (2014). Could behavioral medicine lead the web data revolution?. JAMA.

[8] Allem J, Chu K, Cruz TB, Unger JB (2017). Waterpipe Promotion and Use on Instagram: #Hookah. Nicotine Tob Res.

[9] Krauss MJ, Sowles SJ, Moreno M, Zewdie K, Grucza RA, Bierut LJ, Cavazos-Rehg PA (2015). Hookah-Related Twitter Chatter: A Content Analysis. Prev Chronic Dis.

[10] Grant A, O'Mahoney H (2016). Portrayal of waterpipe (shisha, hookah, nargile) smoking on Twitter: a qualitative exploration. Public Health.

[11] Myslín M, Zhu S, Chapman W, Conway M (2013). Using twitter to examine smoking behavior and perceptions of emerging tobacco products. J Med Internet Res.

[12] Davis CA, Varol O, Ferrara E, Flammini A, Menczer F (2016). Botornot: A system to evaluate social bots.

[13] Ferrara E, Varol O, Davis C, Menczer F, Flammini A (2016). The rise of social bots. Commun ACM.

[14] Allem J, Ferrara E (2016). The Importance of Debiasing Social Media Data to Better Understand E-Cigarette-Related Attitudes and Behaviors. J Med Internet Res.

[15] Primack B, Carroll M, Shensa A, Davis W, Levine M (2016). Sex differences in hookah-related images posted on tumblr: a content analysis. J Health Commun.

[16] Chu Z, Gianvecchio S, Wang H, Jajodia S (2012). Detecting Automation of Twitter Accounts: Are You a Human, Bot, or Cyborg?. IEEE Transactions on Dependable and Secure Computing.

[17] Hutto C, Gilbert E (2015). VADER: A Parsimonious Rule-based Model for Sentiment Analysis of Social Media Text.

[18] Mohammad S, Kiritchenko S, Sobhani P, Zhu X, Cherry C (2016). Detecting Stance in Tweets using Support Vector Machines.

[19] Gou L, Zhou M, Yang H (2014). KnowMeShareMe: understanding automatically discovered personality traits from social mediauser sharing preferences.

[20] Mohammad S (2012). #Emotional tweets.

[21] Porter M (1980). An algorithm for suffix stripping. Program.

[22] Manning CD, Surdeanu M, Bauer J, Finkel J, Bethard SJ, McClosky D (2014). The Stanford CoreNLP Natural Language Processing Toolkit.

[23] Russell J (1980). A circumplex model of affect. J Pers Soc Psychol.

[24] Bradley M, Lang P (1999). NIMH Center for the Study of Emotion and Attention.

[25] Pechmann C, Pan L, Delucchi K, Lakon CM, Prochaska JJ (2015). Development of a Twitter-based intervention for smoking cessation that encourages high-quality social media interactions via automessages. J Med Internet Res.

[26] Pechmann C, Delucchi K, Lakon Cm, Prochaska JJ (2017). Randomised controlled trial evaluation of Tweet2Quit: a social network quit-smoking intervention. Tob Control.

[27] Allem JP, Escobedo P, Chu K, Soto DW, Cruz TB, Unger JB (2017). Campaigns and counter campaigns: reactions on Twitter to e-cigarette education. Tob Control.

[28] Naslund JA, Kim SJ, Aschbrenner KA, McCulloch LJ, Brunette MF, Dallery J, Bartels SJ, Marsch LA (2017). Systematic review of social media interventions for smoking cessation. Addict Behav.

